# Seroepidemiology of peste des petits ruminants virus in small ruminants in selected districts in Northwest Ethiopia

**DOI:** 10.1002/vms3.994

**Published:** 2022-11-11

**Authors:** Habtamu Abesha, Yechale Teshome, Yeshwas Ferede Alemu, Haileyesus Dejene, Zewdu Seyoum Tarekegn, Ayalew Assefa

**Affiliations:** ^1^ Metekel Zone Agriculture and Rural Development Office Metekel Ethiopia; ^2^ College of Agriculture and Environmental Science School of Animal Science and Veterinary Medicine Bahir Dar University Bahir Dar Ethiopia; ^3^ Department of Veterinary Epidemiology and Public Health College of Veterinary Medicine and Animal Sciences University of Gondar Gondar Ethiopia; ^4^ Department of Veterinary Paraclinical Studies College of Veterinary Medicine and Animal Sciences University of Gondar Gondar Ethiopia; ^5^ International Livestock Research Institute (ILRI) Addis Ababa Ethiopia

**Keywords:** Ethiopia, peste des petits ruminants, seroprevalence, small ruminants

## Abstract

**Background:**

Peste des petits ruminants (PPR) is one of the most severe diseases of small ruminants, causing the loss of millions of dollars annually. A cross‐sectional study was conducted to determine the seroepidemiology of peste des petits ruminants virus (PPRV) in unvaccinated sheep and goats in selected districts in Northwest Ethiopia.

**Objectives:**

The study was designed to investigate the epidemiology of PPRV in unvaccinated sheep and goats and risk factors in the study areas.

**Methods:**

A multi‐stage sampling was used to select study districts, villages and households with a random sampling approach. Study animals (403 sheep and goats) older than 5 months were selected with a systematic random sampling approach. From the animals, blood samples were aseptically collected and PPRV antibodies from the serum were analysed with enzyme‐linked immunosorbent assay (ELISA).

**Results:**

The overall seroprevalence of antibodies to PPRV was 32.5% in both species. It was higher in goats with a prevalence of 34.7% than in sheep (28.3%). District, herd size, sex, animal origin and grazing management were significantly associated with seropositivity of animals to PPRV antibodies. If an animal was from the Dangur district, it had 2.6 times higher chance of being positive than in the Dibati district (OR = 2.6, *p* = 0.01 and 95% CI = 1.2– 5.6). Herd size was also significantly correlated with the seropositivity with (OR = 4, *p* = 0.001, and 95% CI = 1.8–9). Also, male animals had 1.7 times higher chance of being positive than females. Further, if an animal comes from the market, it has 2.7 times higher chance of being positive compared to animals born and raised on the farm.

**Conclusions:**

The seropositivity indicates that the disease is circulating in the study areas. Hence, preventive approaches, like vaccination campaigns and strict biosecurity measures, are highly advised to avoid the catastrophic impact of the diseases if an outbreak occurs.

## Graphical abstract

- The overall seroprevalence of antibodies to PPRV was 32.5% in sheep and goats of Metekel zone, Northwest Ethiopia.
- District‐wise, the seroprevalence of the virus was 46% in Pawe, 34% in Dangur, 26% in Mandura and 19% in Dibati.
- Study district, herd size, sex, animal origin and grazing management were significantly associated with seropositivity of animals to PPRV antibodies.

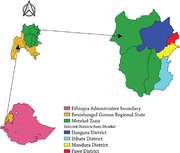

## 1 INTRODUCTION

Sheep and goats are valuable livestock for food and nutrition security for smallholder farmers because of their high reproductive capacity, excellent adaptation to arid environments and low budget price to start a farm. Following population growth, urbanisation and improved living standards, the demand for animal‐derived food is increasing, and as a result, there is a need for sustainable intensification in the small ruminant sector (Markos Tibbo, 2006). Unfortunately, various challenges, such as infectious diseases, affect the production of sheep and goats worldwide and in Africa. Among the diseases affecting sheep and goat production, peste des petits ruminants (PPR) is one of the most severe diseases, with high morbidity and mortality rates (Baron et al., 2016). It is a viral disease characterised by profuse nasal discharge, coughing, stomatitis, fever and diarrhoea in infected animals (Pradère, 2014). It is a transboundary animal disease causing a substantial economic burden, especially in Africa (Diallo, 2006).

The virus was first reported in the Ivory Coast, West Africa, in 1942 and has spread to other areas (FAO, 2009). It is classified under the family *Paramyxoviridae* and the genus *morbillivirus*, closely related to the rinderpest virus (Sen et al., 2010). Recently it was renamed Small Ruminant Morbillivirus (SRMV). In Ethiopia, clinical signs indicative of possible PPRV infection in goatherds in the Afar were first noted in 1977 in goatherds of the Afar regional state but confirmed for the first time in Ethiopia with cDNA probes to have been peste des petits ruminants in 1991 in holding grounds found in Addis Ababa (Roeder et al., 1994). Since then, it has affected the country's pastoral and highland sheep and goat farmers, causing mortalities and poor production potentials of sheep and goats. Reports indicate that it has a seroprevalence of 38% in Afar, 4.6% in Amhara, 8% in Benishangul Gumuz and 1.7% in the Oromia regional state (Abraham et al., 2005; Delil et al., 2012; Megersa et al., 2011; Waret‐Szkuta et al., 2008).

Ethiopia established a plan for the progressive control of PPR that relies on lessons from the eradication of rinderpest. A progressive control program based on continuous immunisation of all susceptible ruminants is not feasible. Thus, epidemiologically targeting endemic regions and high‐risk zones is crucial. Despite the spread of PPRV to previously free locations, few studies have been performed that illustrate the epidemiology of PPRV in Ethiopia (Abraham et al., 2005; Delil et al., 2012; Megersa et al., 2011; Waret‐Szkuta et al., 2008). In many parts of Ethiopia, including the Benishangul Gumuz, where small ruminant production is highly practiced, the disease's distribution remains mostly unstudied. Hence, new epidemiological studies are required to support the FAO plan to eradicate the disease in 2030 nationally and internationally. Therefore, the study aimed to investigate the epidemiology of PPRV in unvaccinated sheep and goats and their associated predictor variables in selected districts of Northwest Ethiopia.

## 2 MATERIALS AND METHODS

### 2.1 Study area

**FIGURE 1:**
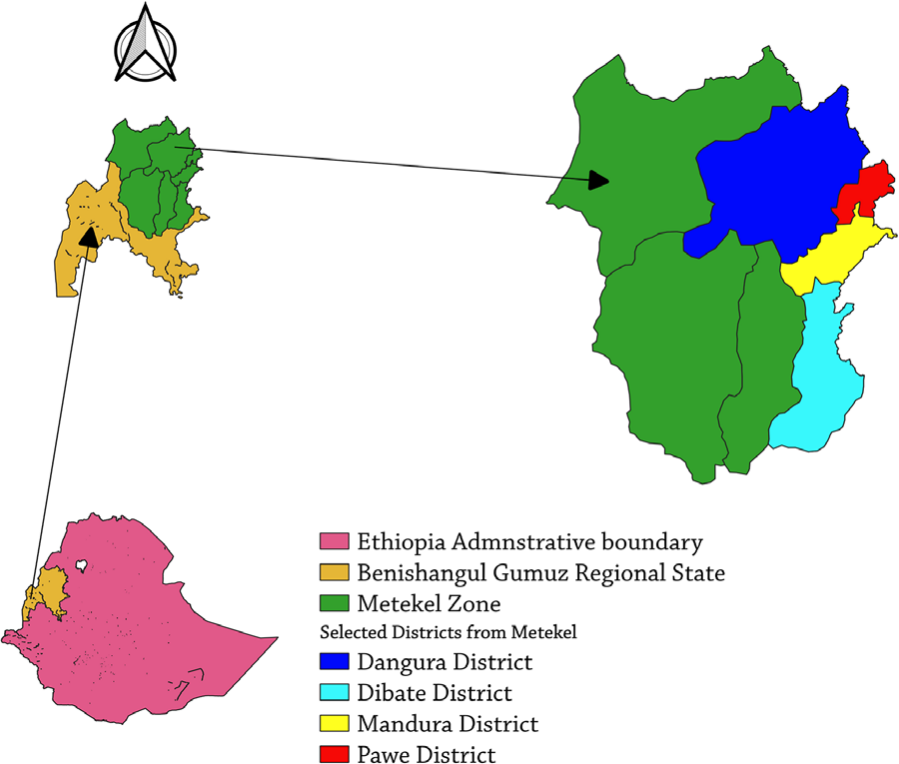
Location map of the study districts

The study was carried out in districts found in Debati, Dangur, Mandura and Pawe districts of the Benishangul Gumuz region, Northwest Ethiopia. The study districts included the valley of Blue Nile located in the Northern part of the Benishangul Gumuz region, which encompasses the lowlands of the Awi zone of the North‐Western Amhara region (CSA, 2016) (Figure 1). Metekel zone is classified as 82% lowland, 10% midland and 8% highland, with an average rainfall of 1275 mm per annum and an altitude range of 500–2731 m above sea level. 55% of the region's total land area is covered with vegetation and forests. The average temperature ranges from 16.2°C to 32.5°C, with an annual mean rainfall of 1600 mm (Wagino & Amanuel, 2021).

### 2.2 Study population

The study populations were sheep and goats of Gumuz goat breed kept under the traditional farming system with no vaccination history against PPR. Moreover, small ruminants younger than 5 months were excluded from sampling to avoid seropositivity due to maternal antibodies as a confounder. The age of the animals was categorised as young (less than 3 years old) and adult (greater than 3 years old).

### 2.3 Study design and sample size determination

The study design was cross‐sectional, and data collection was conducted from November 2019 to July 2020. The sample size was calculated using the formula described by Thrusfield and Christley (2018). Statistical considerations during sample size determination include a 95% confidence interval and 5% desired absolute precision.

$$n=\frac{1.96^{2}P_{\exp}\left(1-P_{\exp}\right)}{d^{2}},$$

Hence, 384 small ruminants (sheep and goats) were required, but we increased the sample size to 403 to allow for mistakes during the sample preparation and analyses. The sample was divided proportionally to the study district according to their small ruminant population size. Based on that, 121, 91, 111 and 80 samples were randomly drawn from Dangur, Mandura, Pawe and Debati districts.

### 2.4 Sampling strategy

A multi‐stage sampling with four hierarchical units was used as a sampling strategy (Dohoo et al., 2003). Districts bordering Amhara regional state were purposively selected because of high contact frequencies with sheep and goats in trade and/or small ruminants kept by nomadic farmers in these areas. Kebeles (the smallest administrative area in Ethiopia), and households, were sampled in random sampling, while animals were selected with a systematic random sampling approach. In each district, four to six rural kebeles were randomly selected proportionally to the number of kebeles in a district by simple random sampling (lottery method) with 19 rural kebeles. Study animals of both sexes and above 5 months’ age groups of sheep and goats were sampled with systematic random sampling. Besides, a mini semi‐structured questionnaire through interviews was administered by asking small ruminant animal owners willing to participate in the study and collect samples from their animals to collect information on the occurrence of PPR in their her and the typical clinical signs they observed.

### 2.5 Data collection

#### 2.5.1 Serum sample collection and analysis

In order to get serum samples for serological detection, 10 ml of blood was collected from each study animal. The collected blood was allowed to clot by placing the samples at room temperature for two consecutive hours without shaking the samples. Then samples were stored horizontally for 3–4 h and serum was separated from the vacutainer tube to cryovials and stored at –20°C until analysed. Samples were analysed using a competitive enzyme‐linked immunosorbent assay (c‐ELISA) test kit (IDScreen^®^ PPR Competition, Montpellier, France) following the manufacturer's instructions.

### 2.6 Data management and analysis

The data obtained was cleared, filtered and coded in MS Excel and analysed using STATA version 14 for statistical analysis (STATA, 2014). Logistic regression was used to quantify the association between the predictor variables and seroprevalence of PPRV antibodies with variables district, species, sex, age, herd size, origin and grazing management. Univariable logistic regression analysis was conducted to reduce the non‐important variables with *p* = 0.25, further model development with stepwise selection in multiple logistic regressions to adjust for confounding and determine their independent effect on PPR seropositivity. A *p* value less than 0.05 was used to indicate a significant level.

## 3 RESULTS

### 3.1 Descriptive results

In four districts of the Metekel zone (Dangur, Mandura, Pawe and Dibati), 265 goats and 138 sheep were sampled for serological analysis (Table 1). A brief questionnaire was introduced to animal owners to get their responses on the occurrence of PPR in their herd. Based on their response to the clinical signs of the diseases, they classified nasal discharge, diarrhoea, respiratory distress, oral ulcer, lacrimation and abortion are the top signs, respectively.

**TABLE 1:** Descriptive characteristics of study area and animals in Northwest Ethiopia

	Variables	No of examined	No of positive	Proportion
District	Dangur	121	41	33.9
	Mandura	91	24	26.4
	Pawe	111	51	45.9
	Dibati	80	15	18.8
	**Total**	**403**	**131**	**32.5**
Species	Goat	265	92	34.7
	Sheep	138	39	28.3
	Total	403	131	32.5
Sex	Male	228	82	35.9
	Female	175	49	28
	Total	403	131	32.5
Age	Adult (>3 years)	230	78	33.9
	Young (<3 years)	173	53	30.6
	Total	403	131	32.5

### 3.2 Seroprevalence of antibodies to PPRV

Of the total samples collected, 32.5% (131/403) were positive for PPR virus antibodies. District‐wise, a relatively higher seroprevalence of PPR was observed in Pawe at 45.9%, compared with Dangur at 33.9%, Mandura at 26.4% and Dibati at 18.8%. The seroprevalence was higher in goats (34.7%) than in sheep (28.3%). Age‐wise, 33.9% of adults and 30.6% of young animals were positive (Table 1).

### 3.3 Association between seropositivity and risk factors

When modelled with univariable logistic regression, study districts, herd size, origin of animals, and grazing management type were significantly associated with seropositivity (Table 2). After removing variables that have *p* > 0.25 from the univariable logistic regression analysis, the final model for multivariable logistic regression analysis containing variables like sex, animal origin, herd size, animal origin, and grazing management type was fitted.

**TABLE 2:** Risk factors associated seropositivity to PPRV in Northwest Ethiopia (univariable logistic regression analysis)

**Variables**	**Groups**	**Crude OR**	***p* Value**	**95% CI**
**Herd size**	Small (<30)	Reference		
	Medium (30–60)	1.8	0.019*	1.1–4.4
	Large (>60)	6.4	0.001***	3.4–12.2
**Districts**	Dibati	Reference		
	Dngur	2.2	0.021*	1.1–4.4
	Mandura	1.6	0.24	0.7–3.2
	Pawe	3.7	0.001***	1.9–7.2
**Species**	sheep	Reference		
	Goat	1.4	0.19	0.9–2.1
**Sex**	Female	1	.	.
	Male	1.4	0.09	0.9–2.2
**Age category**	Adult	Reference		
	Young	0.9	0.49	0.6–1.3
**Animal origin**	Born in	Reference		
	Brought in	2.5	0.001***	1.6–3.8
**Grazing management**	Private land	Reference		
	Communal land	2.5	0.001***	1.6–4

* significant at alpha value less than or equal to 0.05.

** significant at alpha value less than or equal to 0.01.

*** significant at alpha value less than or equal to 0.001.

Districts, herd size, sex, animal origin and grazing management were significantly associated with seropositivity. An animal from the Dangur district had 2.6 times more chance of being seropositive than the reference group Dibati district (OR = 2.6, *p* = 0.01 and 95% CI 1.2–5.6). If an animal comes from the Mandura district, it had a chance of 1.6 times higher than Dibati, which was not statistically significant (OR = 1.6, *p* = 0.25 and 95% CI = 1.2–5.6). Further, animals from Pawe had a 4.3 times higher chance of being seropositive than the reference (OR = 4.3, *p* = 0.001 and 95% CI = 2–9).

Furthermore, herd size was also significantly correlated with the seropositivity of animals to PPRV antibodies. If animals were from large herd sizes, they had an odd of 4 times higher chance of being positive than the reference small herd size (OR = 4, *p* = 0.001, and 95% CI = 1.8–9). Whereas medium herd size had a numerically higher chance of being positive than small herd size, the difference was not statistically significant.

Sex of animals was also associated with seropositivity to PPRV antibodies, in which male animals had a higher chance of being positive than females. If an animal is male, it has a 70% higher chance of being positive than females, in which the difference is statistically significant (Or = 1.7, *p* = 0.029 and 95% CI = 1.1–2.8). The origin of animals (bought from the market or born and raised in the household) was the other risk factor with a significant association with seropositivity to PPRV antibodies. If an animal comes from the market, it has a 2.7 times higher chance of being positive compared to animals born and raised on the farm (OR = 2.7, *p* = 0.001, and 95% CI = 1.6–4.4). Grazing management was also significantly associated with seropositivity. If an animal was allowed to graze in communal grasslands, it had a 2.3 times higher chance of being positive (OR = 2.3, *p* = 0.01 and 95% CI = 1.2–4.2) (Table 3).

**TABLE 3:** Risk factors associated seropositivity to PPRV in Northwest Ethiopia (multivariable logistic regression analysis)

**Variables**	**Groups**	**Adjusted OR**	***p* Value**	**95% CI**
Districts	Dibati	Reference		
	Dangur	2.6	0.01**	1.2–5.6
	Mandura	1.6	0.25	0.7–3.6
	Pawe	4.3	0.001***	2–9
Herd size	Small (<30)	Reference		
	Medium (30–60)	1.2	0.63	0.6–2.2
	Large (>60)	4	0.001***	1.8–9
Sex	Female	Reference		
	Male	1.7	0.029**	1.1–2.8
Animal origin	Born in	Reference		
	Brought in	2.7	0.001***	1.6–4.4
Grazing management	Private land	Reference		
	Communal land	2.3	0.01**	1.2–4.2

* significant at alpha value less than or equal to 0.05.

** significant at alpha value less than or equal to 0.01.

*** significant at alpha value less than or equal to 0.001.

## 4 DISCUSSION

For effective control and eradication of PPR, timely vaccination of susceptible populations is recommended (Baron et al., 2011). Also, herd‐level immunity detection and epidemiological surveys help understand the disease status and design effective disease control programs.

In this study, we reported an overall prevalence of 32.5% PPRV antibodies in both sheep and goats. The seroprevalence detected was higher than previous reports (Gebre et al., 2018) in Southwest Ethiopia reported a seroprevalence of 2.1% (Fentie et al., 2018, 2017) in the Amhara Region with an overall prevalence of 18.3% and (Megersa et al., 2011) in the pastoral and agropastoral system in Ethiopia with a prevalence of 7.3% in sheep but higher prevalence of 42% in goats. In contrast to our findings, (Gizaw et al., 2018) and (Yalew et al., 2019) have reported a higher prevalence of PPR in the Afar region and the Asossa zone of Ethiopia, with a prevalence of 41.5% and 75.5%, respectively. The prevalence of PPR observed in the different settings in Ethiopia could be attributed to the differences in management practices, vaccination status and agroecological differences because agroecology, climate and vegetation determine the spread of the virus during an outbreak (Assefa et al., 2021).

Risk factors like herd size, sex, origin and grazing management were significantly associated with seropositivity. Significantly higher seroprevalence was reported in large herd sizes than in medium and small herd sizes. The higher prevalence in larger herds can be due to health management in larger flocks and high disease transmission within the flock, consequently increasing the number of infected animals. Sex‐wise, the prevalence was higher in males than females, which was statistically significant. A higher prevalence in males can be due to higher demands on male animals for meat purposes, driving them to the market and exposing them to a higher infection rate than in females, which are relatively kept at home for breeding purposes. Contrary to our study, a study conducted in Afar regional State indicated that all determinant factors were not associated with seroprevalence of PPR (Gizaw et al., 2018). Also, in research conducted in Nigeria, there were no significant differences in the PPRV seroprevalence between male and female animals (Woma et al., 2016).

Furthermore, the origin of animals was also significantly associated with the origin of animals. The odds of seropositivity were 2.72 times higher in animals bought from the market than those born in the household. Animals from the market have a higher risk of exposure to infection due to the intermingling of animals. Besides, Grazing management also contributes significantly to the seroprevalence of animals. If an animal is managed in communal grazing, it has a 2.22 higher chance of being positive than those contained in private grazing. Grazing management in communal grassland is similar to a market in which animals come in contact to increase the risk of exposure. Similarly, in a study conducted in Southern Ethiopia, Flock size and the recent introduction of new animals to the flock were significantly associated with Sero prevalence (Hailegebreal, 2018).

## 5 CONCLUSIONS

The overall seroprevalence of PPRV in districts in Northwest Ethiopia was 32.5% in unvaccinated sheep and goats. Districts, herd size, sex, animal origin and grazing management were significantly associated with seropositivity of animals to PPRV antibodies. The disease is circulating in the study areas with higher prevalence, indicating the need for coordinated prevention and control plan to reduce the impact of the disease. Hence the application of farm‐level biosecurity measures, broader coverage of vaccination campaigns and awareness creation among farmers is highly recommended to control and eradicate the disease by 2030 as planned by the OIE. Further studies are recommended to identify lineages of the PPR virus circulating in the area.

## AUTHOR CONTRIBUTIONS

**Habtamu Abesha**: investigation; methodology; project administration; resources; supervision; data curation; formal analysis; validation; visualisation; writing – original draft; writing – review & editing. **Yechale Teshome**: methodology; project administration; resources; supervision; data curation; formal analysis; validation; visualisation; writing – original draft; writing – review & editing. **Yeshwas Ferede Alemu**: validation; visualisation; writing – original draft; writing – review & editing. **Haileyesus Dejene**: validation; visualisation; writing – original draft; writing – review & editing. **Zewdu Seyoum Tarekegn**: writing – original draft; writing – review & editing. **Ayalew Assefa**: writing – original draft; writing – review & editing.

## CONFLICT OF INTEREST

We declare that there is no conflict of interest.

## FUNDING STATEMENT

This research doesn't receive financial support from public or private entities.

### ETHICS STATEMENT

The authors confirm that the ethical policies of the journal, as noted on the journal's author guidelines page, have been adhered to and the Ethiopia National Research Ethics Review Guideline for the Care and Use of Animals was followed.

### PEER REVIEW

The peer review history for this article is available at https://publons.com/publon/10.1002/vms3.994.

## Data Availability

Available upon request of the authors.
